# Characteristics of pachychoroid neovasculopathy

**DOI:** 10.1038/s41598-020-73303-w

**Published:** 2020-10-01

**Authors:** Miho Tagawa, Sotaro Ooto, Kenji Yamashiro, Hiroshi Tamura, Akio Oishi, Manabu Miyata, Masayuki Hata, Munemitsu Yoshikawa, Nagahisa Yoshimura, Akitaka Tsujikawa

**Affiliations:** Department of Ophthalmology and Visual Sciences, Kyoto University Graduate School of Medicine, 54 Kawahara-cho Shogoin, Sakyo-ku, Kyoto, 606-8507 Japan

**Keywords:** Macular degeneration, Retinal diseases

## Abstract

Recently, several research groups have reported a newly recognized clinical entity of choroidal neovascularization, termed pachychoroid neovasculopathy. However, its characteristics have yet to be well described. The purpose of this study was to investigate the clinical and genetic characteristics of pachychoroid neovasculopathy regardless of treatment modality. This study included 99 eyes of 99 patients with treatment-naïve pachychoroid neovasculopathy. Mean initial best-corrected visual acuity (BCVA) was 0.20 ± 0.32 logMAR, and did not change (*P* = 0.725) during follow-up period (mean ± SD, 37.0 ± 17.6 months). Subretinal hemorrhage (SRH) (≥ 4 disc areas in size) occurred in 20 eyes (20.2%) during follow-up. Age, initial BCVA, central retinal thickness, SRH (≥ 4 disc areas in size) and treatment (aflibercept monotherapy) were significantly associated with the final BCVA (*P* = 0.024, < 0.001, 0.031, < 0.001, and 0.029, respectively). Multiple regression analysis showed initial BCVA and presence of SRH to be significant predictors of final BCVA (both *P* < 0.001). Polypoidal lesions were more common in the SRH group than in the non-SRH group (85.0% vs 48.1%, *P* = 0.004). There was no significant difference in the frequency of the risk allele in *ARMS2* A69S, *CFH* I62V, *CFH* Y402H between these groups (*P* = 0.42, 0.77, and 0.85, respectively). SRH (29.1% vs 9.1%, *P* = 0.014) and choroidal vascular hyperpermiability (65.5% vs 43.2%, *P* = 0.027) were seen more frequently in the polypoidal lesion (+) group than in the polypoidal lesion (−) group. There was considerable variation in lesion size and visual function in patients with pachychoroid neovasculopathy, and initial BCVA and presence of SRH at the initial visit or during the follow-up period were significant predictors of final BCVA.

## Introduction

Age-related macular degeneration (AMD) is a leading cause of severe visual loss in elderly people^1,2^. Early findings of AMD are extracellular deposits between the retinal pigment epithelium and Bruch’s membrane, including drusen^3,4^. Late AMD is characterized by two types of lesions, i.e., neovascular and dry AMD. The hallmark of neovascular AMD is neovascularization, which causes subretinal hemorrhage and exudative changes. It has been reported that formation of large soft drusen and subsequent hyperpigmentation are associated with evolution of neovascularization in AMD^4^.

Recently, several research groups have reported a newly recognized clinical entity of neovascularization, termed pachychoroid neovasculopathy^5,6^. “Pachychoroid” indicates choroidal characteristics, including a focal or diffuse increase of choroidal thickness and dilation of outer choroidal vessels. It has been reported that pachychoroid-driven spectrum diseases include pachychoroid pigment epitheliopathy^7^, central serous chorioretinopathy (CSC), polypoidal choroidal vasculopathy (PCV)^8^, and pachychoroid neovasculopathy^9–11^. Recently, Miyake et al. showed that pachychoroid neovasculopathy differs from neovascular AMD not only phenotypically but also genetically^6^. Hata et al. reported that the mean concentration of vascular endothelial growth factor (VEGF) in aqueous humor samples was significantly lower in eyes with pachychoroid neovasculopathy than in those with neovascular AMD^12^. Terao et al. found that VEGF-A in the pachychoroid neovascular group was significantly lower than in the neovascular AMD group^13^. These findings suggest that the mechanism of development of neovascularization may differ between pachychoroid neovasculopathy and neovascular AMD, and raise the possibility that the clinical characteristics differ between the two conditions. However, little is presently known about the clinical characteristics of pachychoroid neovasculopathy because previous reports included only small numbers of patients. The purpose of this study was to investigate the clinical and genetic characteristics of pachychoroid neovasculopathy, mainly focused on factors associated with visual prognosis, in a larger group of Japanese patients.‬

## Results

A total of 99 eyes of 99 patients (79 men, 20 women) were included in the study. The characteristics of the participants are summarized in Table 1. Briefly, all patients were Japanese and had a mean age of 69.3 ± 8.1 (range 43–86) years. The mean follow-up duration was 37.0 (range 12–75) months. The mean axial length was 23.36 (range 21.03–25.73) mm.

**Table 1:** Patient baseline characteristics.

Total n = 99 eyes	
Gender (men/women)	79/20
Age (years), mean ± SD (range)	69.3 ± 8.1 (43–86)
Axial length (mm)	23.36 ± 0.91 (21.03–25.73)
Smoking (%)	71 (71.7)
Hypertension (%)	53 (53.5)
Follow-up period (months), mean ± SD (range)	37.0 ± 17.6 (12–75)

Smoking means the number of current and past smokers.

*SD* standard deviation.

**Figure 1:**
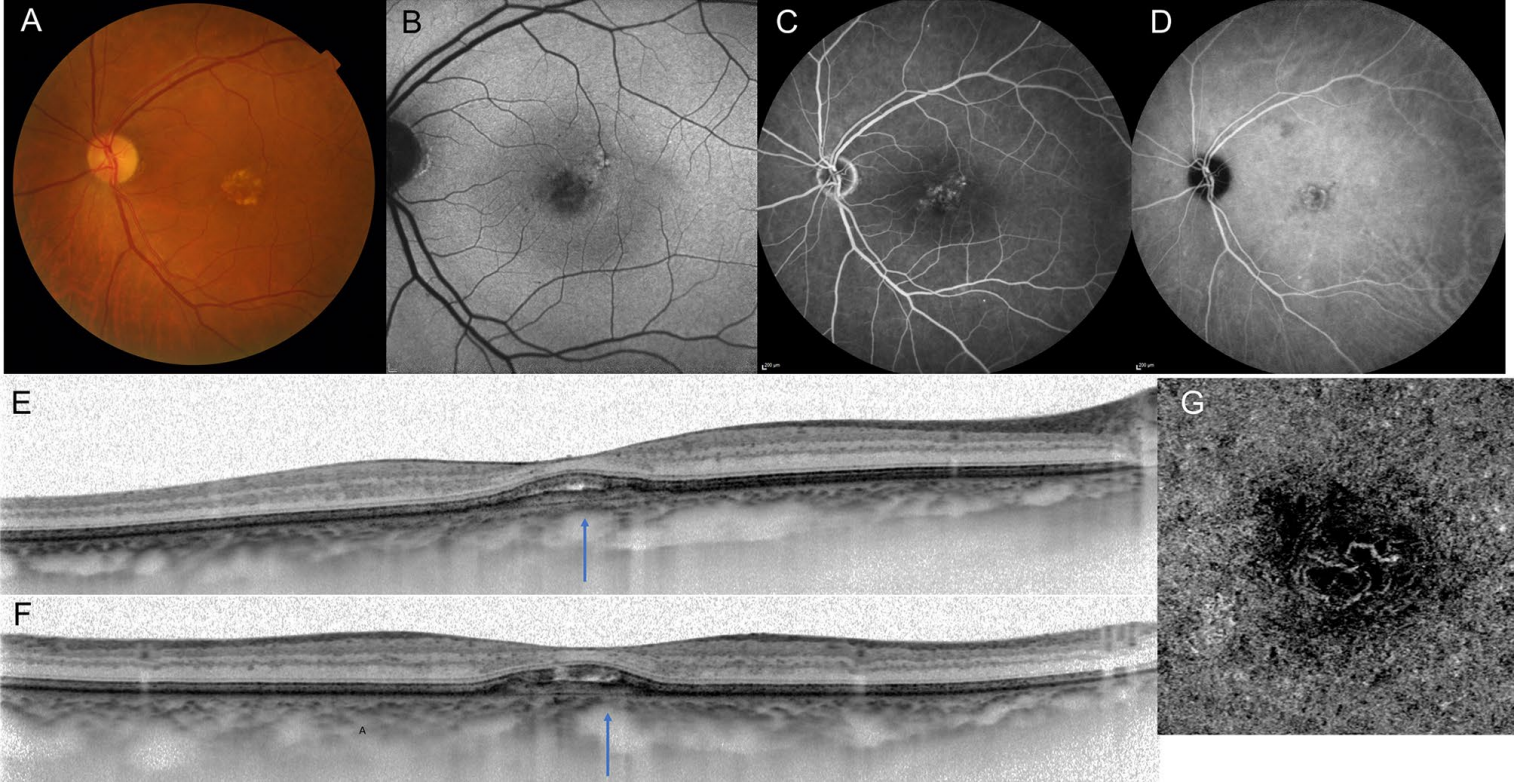
A case of pachychoroid neovasculopathy. Images from a 72-year-old man with visual impairment in the left eye. Best corrected visual acuity (BCVA) was 20/25. (**A**) Color fundus photographs showing reduced fundus tessellation without drusen, and serous retinal detachment. (**B**) Fundus autofluorescence shows granular hypo-autofluorescence. (**C**) Fluorescein angiography image showing leakage suggesting occult choroidal neovascularization (CNV). (**D**) Indocyanine green angiography image showing dilated choroidal vessels. (**E,F**) Enhanced-depth imaging optical coherence tomography (OCT) through the fovea (**D**, horizontal; **E**, vertical) showing dilated large choroidal vessels with obliteration of the choriocapillaris (arrows), retinal pigment epithelium protrusion suggesting a choroidal neovascularization and serous retinal detachment. The subfoveal choroidal thickness is 340 µm. (**F**) OCT angiography enface image showing the presence of CNV.

**Figure 2:**
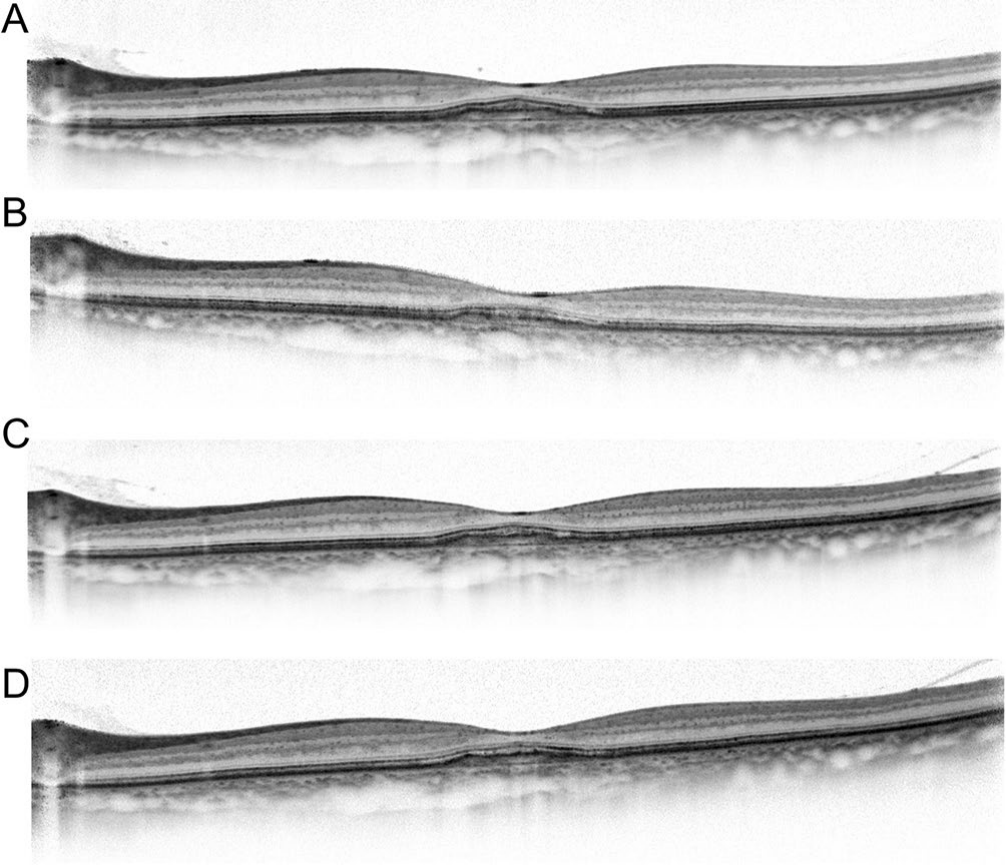
A case of pachychoroid neovasculopathy after treatment. Images of the same patient as in Fig. 1. The patient received three courses of monthly and subsequent bimonthly aflibercept injection for one year. (**A**) Three months after the initial injection. Optical coherence tomography (OCT) showing resolution of serous retinal detachment. Best corrected visual acuity (BCVA) has improved to 20/20. (**B**) Twelve months after the initial injection. OCT shows a dry macula. BCVA has improved to 24/20. (**C**) Twenty-four months after the initial injection. OCT showing no recurrence of exudative changes. BCVA has improved to 30/20. (**D**) Thirty-six months after the initial injection. OCT showing no recurrence of exudative changes. BCVA is maintained as 30/20.

Initial and final conditions are shown in Table 2. At baseline, the mean logarithm of the minimum angle of resolution (logMAR) best-corrected visual acuity (BCVA) was 0.20 (Snellen equivalent 20/30). The mean lesion area and greatest linear dimension (GLD) were 3.01 ± 3.28 mm^2^ and 3241 ± 1819 µm, respectively. The mean central retinal thickness (CRT) and subfoveal choroidal thickness were 321.0 ± 204.4 µm and 307.6 ± 67.4 µm, respectively. Polypoidal lesions were seen in 55 eyes (55.6%) and choroidal vascular hyperpermeability (CVH) in 56 eyes (56.6%). A typical case is shown in Figs. 1 and 2.

**Table 2:** Initial and final conditions.

Total n = 99 eyes	
**Initial conditions**	
BCVA (logMAR), mean ± SD (range)	0.20 ± 0.32 (− 0.176 ~ 1.699)
Lesion area (mm^2^), mean ± SD (range)	3.01 ± 3.28 (0.11 ~ 13.58)
GLD (µm), mean ± SD (range)	3241 ± 1819 (624 ~ 7156)
CRT (µm), mean ± SD (range)	321.0 ± 204.4 (31 ~ 1451)
SFCT (µm), mean ± SD (range)	307.6 ± 67.4 (210 ~ 491)
Polypoidal lesion (%)	55 (55.6)
CVH (%)	56 (56.6)
**At the initial visit and during the follow-up period**	
SRH (≥ 4 disc areas in size) (%)	20 (20.2)
**Final conditions**	
BCVA (logMAR), mean ± SD (range)	0.21 ± 0.39 (− 0.176 ~ 1.523)
CRT (µm), mean ± SD (range)	173.6 ± 91.5 (31 ~ 558)

**Figure 3:**
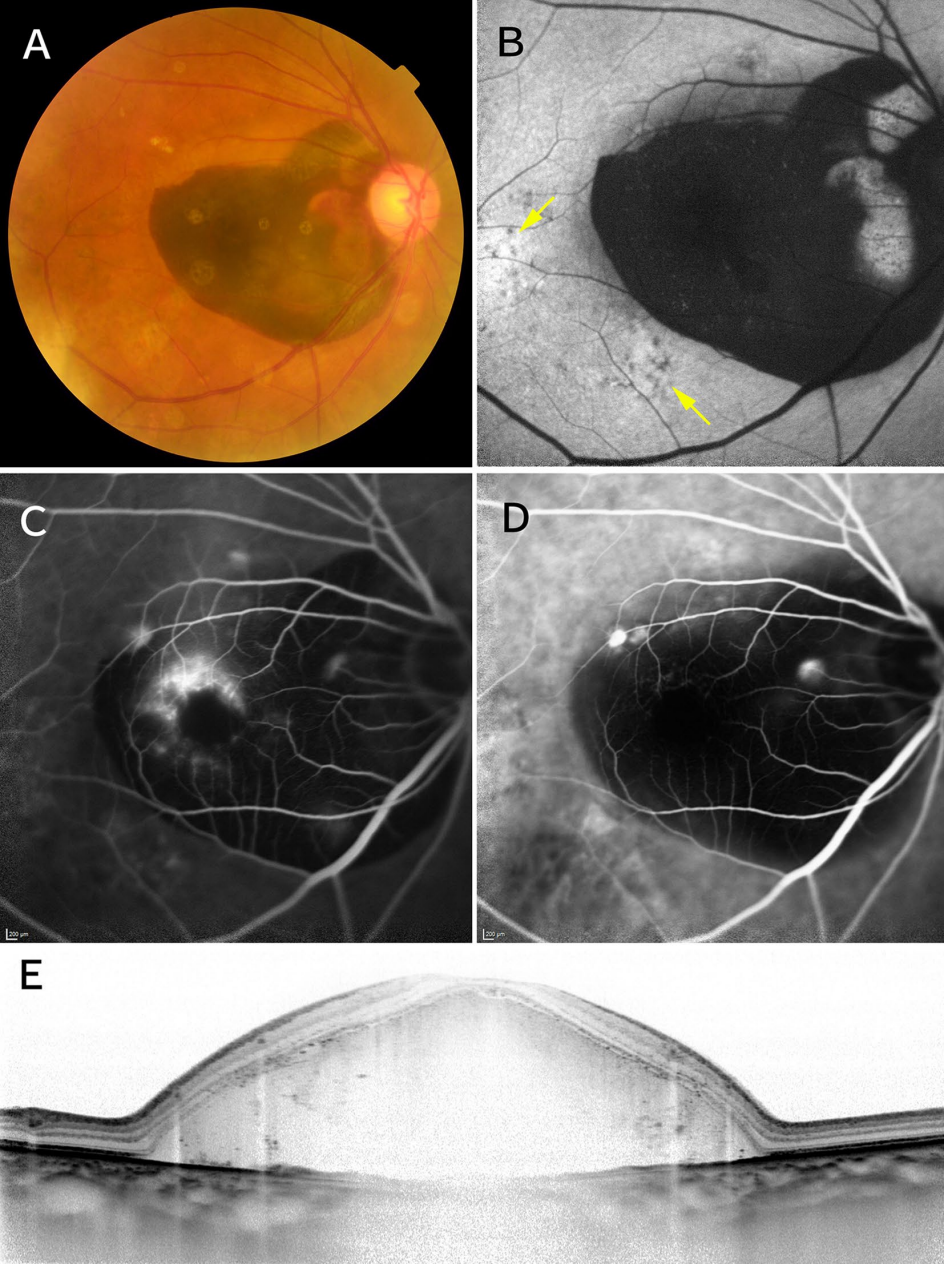
A case of pachychoroid neovasculopathy with subretinal hemorrhage. Images from a 77-year-old man with visual impairment in the right eye. Best-corrected visual acuity was 20/200. (**A**) Color fundus photographs showing subretinal hemorrhage (SRH) ≥ 4 disc areas in size and no drusen. (**B**) Fundus autofluorescence showing retinal pigment epithelium abnormalities (arrows) apart from SRH. (**C**) Fluorescein angiography showing leakage suggesting occult choroidal neovascularization. (**D**) Indocyanine green angiography showing polypoidal lesions. (**E**) Enhanced-depth imaging optical coherence tomography showing dilated large choroidal vessels and massive SRH. The subfoveal choroidal thickness is 305 µm.

**Figure 4:**
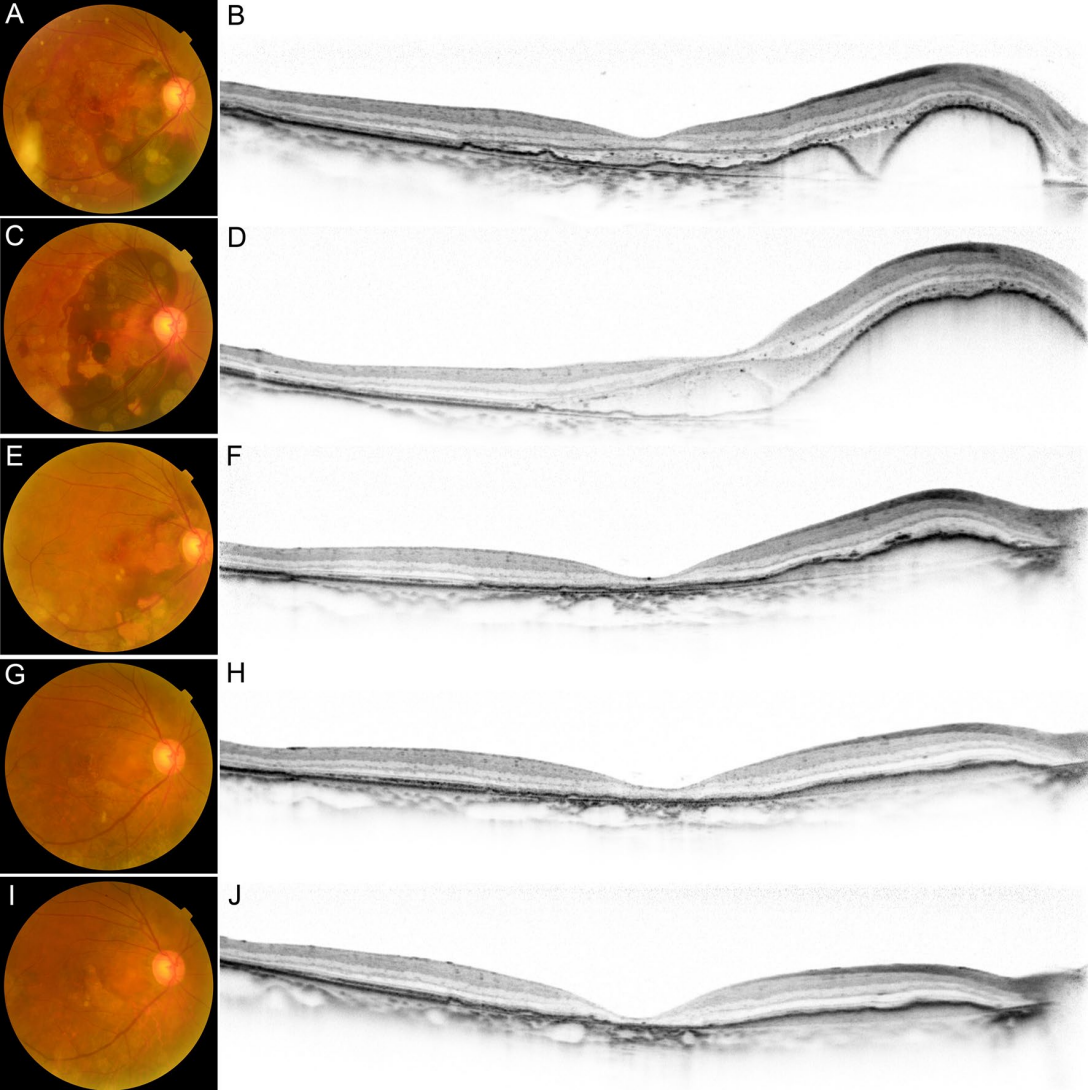
A case of pachychoroid neovasculopathy with subretinal hemorrhage after treatment. Images of the same patient as in Fig. 3. The patient received intravitreous gas and aflibercept injection. (**A,B**) After this treatment, the subretinal hemorrhage (SRH) moved to outside the fovea. Best-corrected visual acuity (BCVA) improved to 20/50. (**C,D**) Two days later, the SRH recurred. BCVA deteriorated to 20/200. The patient received three courses of monthly and subsequent bimonthly aflibercept injection for one year. (**E,F**) Three months later, optical coherence tomography (OCT) showing reduction of SRH. BCVA was 20/100. (**G,H**) Twelve months after the initial injection. OCT showing resolution of SRH. BCVA has improved to 20/40. (**I,J**) Twenty-eight months after the initial injection. OCT showing dry macula but disruption of ellipsoid zone band. BCVA is 20/40.

*SD* standard deviation; *BCVA* best-corrected visual acuity; *GLD* greatest linear dimension; *CRT* central retinal thickness; *SFCT* subfoveal retinal thickness; *CVH* choroidal vascular hyperpermeability; *SRH* subretinal hemorrhage;

At the initial visit, subretinal hemorrhage (SRH) (≥ 4 disc areas in size^14^) was detected in 9 eyes. During the follow-up period, SRH (≥ 4 disc areas in size) occurred in 11 eyes. Therefore, SRH (≥ 4 disc areas in size) was seen in total 20 eyes (20.2%) within the follow-up period, and 17 eyes (85%) of these 20 eyes had polypoidal lesions (Figs. 3 and 4).

At the final visit, there was no statistically significant change in mean logMAR BCVA (0.21 ± 0.39, *P* = 0.725) but there was a significant decrease in CRT (173.6 ± 91.5 µm, *P* < 0.001) when compared with baseline.

Table 3 shows the values of the single regression analysis of potential predictors (initial conditions and during follow up period) of final BCVA. Age, initial BCVA, CRT, SRH (≥ 4 disc areas in size), aflibercept monotherapy were significantly associated with the final BCVA (*P* = 0.024, < 0.001, 0.031, < 0.001, and 0.029 respectively). In multiple regression analysis, only initial BCVA and presence of SRH (≥ 4 disc areas in size) were significantly associated with final BCVA (both *P* < 0.001).

**Table 3:** Factors associated with final best-corrected visual acuity.

Factors associated with final BCVA	*P* value
Gender	0.284
Age	0.024
Smoking	0.662
Axial length	0.367
Hypertension	0.718
Initial BCVA (logMAR)	< 0.001
Lesion area	0.067
GLD	0.071
CRT	0.031
SFCT	0.656
Polypoidal lesion	0.458
CVH	0.248
SRH (≥ 4 disc areas in size) at the initial visit and during the follow-up period	< 0.001
Treatment (aflibercept monotherapy)	0.029
Treatment (initial PDT)	0.260

*BCVA* best-corrected visual acuity; *GLD* greatest linear dimension; *CVH* choroidal vascular hyperpermeability; *SRH* subretinal hemorrhage; *CRT* central retinal thickness; *SFCT* subfoveal choroidal thickness; *PDT* photodynamic therapy.

The presence of SRH was a significant factor with regard to visual prognosis. Therefore, we divided the eyes with pachychoroid neovasculopathy into two groups, i.e., an SRH group (SRH ≥ 4 disc areas in size seen at the initial visit or during the follow-up period; n = 20) and a non-SRH group (n = 79; Table 4). There were no significant differences in gender and in the mean patient age (*P* = 0.517, *P* = 0.053, respectively). However, the mean initial GLD was larger in the SRH group than in the non-SRH group (4698 ± 2302 µm vs 2909 ± 1519 µm, *P* = 0.005). At baseline, there were no significant differences in BCVA between the two groups (*P* = 0.455). At the final visit, the mean BCVA was worse in the SRH group than in the non-SRH group (0.55 ± 0.60 vs 0.12 ± 0.26, *P* = 0.005). Polypoidal lesions were seen more often in the SRH group than in the non-SRH group (85.0% vs 48.1%, *P* = 0.004) at the initial visit.

**Table 4:** Characteristics of pachychoroid neovasculopathy in subretinal hemorrhage group and non- subretinal-hemorrhage group.

	SRH group (n = 20)	Non-SRH group (n = 79)	*P* value
Gender (men/women)	17/3	62/17	0.517**
Age (years), mean ± SD	66.2 ± 9.5	70.1 ± 7.6	0.053*
Axial length (mm)	23.37 ± 0.91	23.36 ± 0.91	0.959*
Hypertension (%)	8 (40.0)	45 (57.0)	0.174**
Smoking (%)	15 (75.0)	56 (70.9)	0.775**
Follow-up period (months), mean ± SD	35.0 ± 18.9	38.0 ± 17.4	0.525*
**Initial conditions**			
BCVA (logMAR), mean ± SD	0.27 ± 0.55	0.18 ± 0.24	0.455*
Lesion area (mm^2^), mean ± SD	2.48 ± 3.21	3.50 ± 3.30	0.363*
GLD (µm), mean ± SD	4698 ± 2302	2909 ± 1519	0.005*
CRT (µm), mean ± SD	417.1 ± 365.8	296.7 ± 130.1	0.163*
SFCT (µm), mean ± SD	310.3 ± 53.1	306.9 ± 70.9	0.845*
Polypoidal lesion (%)	17 (85.0)	38 (48.1)	0.004**
CVH (%)	11 (55.0)	45 (57.0)	0.211**
**Final conditions**			
BCVA (logMAR), mean ± SD	0.55 ± 0.60	0.12 ± 0.26	0.005*
CRT (µm), mean ± SD	153.2 ± 99.7	178.7 ± 89.3	0.266*

*SRH* subretinal hemorrhage; *SD* standard deviation; *BCVA* best-corrected visual acuity; *GLD* greatest linear dimension; *CRT* central retinal thickness; *SFCT* subfoveal choroidal thickness; *CVH* choroidal vascular hyperpermeability.

*Unpaired t-test, **Chi-square test.

We also divided the eyes with pachychoroid neovasculopathy into two groups to by the presence of polypoidal lesion, i.e., a polypoidal lesion (+) group (n = 55) and a polypoidal lesion (−) group (n = 44; Table 5). The frequencies of SRH (≥ 4 disc areas in size**)** and CVH were higher in the polypoidal lesion (+) group (*P* = 0.014, *P* = 0.027, respectively). Lesion area was smaller in the polypoidal lesion (+) group (*P* = 0.010). There was no significant difference in final BCVA between these two groups. Moreover, in the polypoidal lesion (+) group, we further investigated the portion of peripapillary lesion. Among 55 eyes, seven eyes (12.7%) had peripapillary lesions. The presence of peripapillary polypoidal lesion and the presence of non-peripapillary polypoidal lesion had no association with final BCVA (*P* = 0.181, *P* = 0.595, respectively).

**Table 5:** Characteristics of Pachychoroid Neovasculopathy in Polypoidal Lesion (+) Group and without Polypoidal Lesion (−) Group.

	Polypoidal lesion (+) group (n = 55)	Polypoidal lesion (−) group (n = 44)	*P* value
Gender (men/women)	43/12	37/7	0.458**
Age (years), mean ± SD	69.3 ± 8.5	69.3 ± 7.4	0.996*
Axial length (mm)	23.42 ± 1.04	23.28 ± 0.72	0.921*
Hypertension (%)	31 (56.4)	22 (50.0)	0.528**
Smoking (%)	37 (67.3)	34 (77.3)	0.272**
Follow-up period (months), mean ± SD	37.5 ± 18.3	37.3 ± 16.6	0.951*
**Initial conditions**			
BCVA (logMAR), mean ± SD	0.16 ± 0.30	0.24 ± 0.34	0.248*
Lesion area (mm^2^), mean ± SD	2.20 ± 2.42	3.99 ± 3.87	0.010*
GLD (µm), mean ± SD	3245 ± 1989	3204 ± 1579	0.913*
CRT (µm), mean ± SD	317.7 ± 243.0	323.2 ± 138.9	0.895*
SFCT (µm), mean ± SD	297.7 ± 68.2	319.2 ± 63.9	0.116*
SRH (≥ 4 disc areas in size) (%) (at the initial visit and the during follow-up period)	16 (29.1)	4 (9.1)	0.014**
CVH (%)	36 (65.5)	19 (43.2)	0.027**
**Final conditions**			
BCVA (logMAR), mean ± SD	0.21 ± 0.40	0.21 ± 0.38	0.944*
CRT (µm), mean ± SD	178.2 ± 107.3	168.5 ± 65.5	0.606*

*SD* standard deviation; *BCVA* best-corrected visual acuity; *GLD* greatest linear dimension; *CRT* central retinal thickness; *SFCT* subfoveal choroidal thickness; *SRH* subretinal hemorrhage; *CVH* choroidal vascular hyperpermeability.

*Unpaired t-test, **Chi-square test.

During the follow-up period, 8 patients were observed without any treatment due to no exudative changes. Ninety-one eyes were treated by anti-VEGF injection, photodynamic therapy (PDT), or pars plana vitrectomy (PPV). Sixty-nine eyes were treated by anti-VEGF (ranibizumab and/or aflibercept) monotherapy and 14 eyes were treated by PDT. Among 69 eyes treated by anti-VEGF monotherapy, 30 eyes were treated with intravitreous aflibercept (IVA) monotherapy. Among 14 eyes treated by PDT, eight patients were treated initially by PDT combined with anti-VEGF injection and 6 patients were treated by PDT as a rescue therapy. PPV was performed in 6 eyes and intravitreal sulfur hexafluoride (SF_6_) gas and anti-VEGF injection were administered in 4 eyes. All of them were in the SRH group and had polypoidal lesions.

Table 6 shows the genetic characteristics of the 92 patients with pachychoroid neovasculopathy who underwent genotyping. The frequency of the T allele in *ARMS2* A69S was 46.7% in this group. There was no significant difference in the frequency of the T allele in *ARMS2* A69S between the SRH group and the non-SRH group (52.9% vs 45.3%, *P* = 0.42). The frequency of the A allele in *CFH* I62V was 25.5%. The frequency of the C allele in *CFH* Y402H was 10.9%. There were no significant differences in the frequency of these alleles between the SRH group and the non-SRH group (*P* = 0.77 and *P* = 0.85, respectively).

**Table 6:** Genetic characteristics.

	Data from	n	ARMS2 A69S					CFH I62V					CFH Y402H				
			GG	GT	TT	T-allele frequency (%)	P	AA	AG	GG	A-allele frequency (%)	P	TT	CT	CC	C-allele frequency (%)	P
Pachychoroid neovasculopathy	Current study	92	28	42	22	46.7	–	9	29	54	25.5	–	75	14	3	10.9	–
SRH +	Current study	17	4	8	5	52.9	0.42	1	6	10	23.5	0.77	13	4	0	11.8	0.85
SRH−		75	24	34	17	45.3		8	23	44	26.0		62	10	3	10.7	
Normal (Japanese)	Nagahama	3248	1312	1499	435	36.5	–	546	1538	1162	40.5	–					
Pachychoroid neovsculopathy (Japanese)	Miyake et al	39	11	16	12	51.3	0.029	8	16	15	41.0	0.013					
Neovascular AMD (Japanese)		161	24	64	71	64.8		17	48	96	25.5						
Neovascular AMD (Japanese)	Arakawa et al	1536	–	–	–	57.4	–	–	–	–	27.1	–					
Pachychoroid neovasculopathy (Caucasian)	Dansingani et al	50	–	–	–	41	0.672						–	–	–	46	0.0236
Neovascular AMD (Caucasian)		51	–	–	–	44							–	–	–	63	

*AMD* age-related macular degeneration; *SRH* subretinal hemorrhage.

## Discussion

Pachychoroid neovasculopathy is a recently recognized clinical entity of neovascularization, and little is known about its characteristics. In the current study, we investigated the clinical and genetic characteristics of 99 eyes with pachychoroid neovasculopathy. Many cases had relatively small lesions and good BCVA, while some had large lesions and poor BCVA. Our study demonstrated that the initial BCVA and the presence of SRH at the initial visit or during the follow-up period were significant predictors of the final BCVA and that eyes with SRH had polypoidal lesions more often.

Our study demonstrated that men with pachychoroid-related disease substantially outnumbered women, which is also observed in pachychoroid GA^15^. This trend might be explained by the relatively high prevalence of CSC among men reported in several studies^16,17^. The mean subfoveal choroidal thickness in our study (307.6 µm) is comparable with that in the study by Miyake et al. (310 µm)^6^, Pang et al. (244–336 µm)^5^, and in the study of pachychoroid GA (353 µm)^15^. CVH was found in 56.6% of eyes with pachychoroid neovasculopathy, which is consistent with the value reported by Miyake et al. (53.8%)^6^ and that in the study of pachychoroid GA (47.4%)^15^. In the current study, CVH was more frequently seen in the polypoidal lesion (+) group. Sasahara et al. reported that CVH occurred more frequently in PCV than that in AMD, which is similar to our results^18^.

At the initial visit and during the follow-up period, SRH (≥ 4 disc areas in size) occurred in 20 eyes, and many of them had polypoidal lesions. Among these eyes, 9 (45%) had SRH (≥ 4 disc areas in size) at the initial visit. The presence of polypoidal lesion and SRH at the initial visit and during the follow-up period were associated with developing SRH. There has been no report on the occurrence of SRH in pachychoroid neovasculopathy. In a study by Hirami et al., SRH was seen in 28 (30.8%) of 91 eyes with PCV after PDT and bleeding resulted in a vitreous hemorrhage in 6 eyes^19^. In the current study, SRH was seen in 20.2% of eyes and PPV was performed in 6 eyes. There are several possible explanations for the relatively lower frequency of SRH in our study. First, 55.6% of the eyes with pachychoroid neovasculopathy had polypoidal lesions whereas all eyes with PCV had these lesions. Polypoidal lesions are thought to be the source of massive hemorrhage^19^. Second, PDT monotherapy was performed in the study by Hirami et al.^19^, whereas anti-VEGF monotherapy and PDT combined with anti-VEGF injection, which may cause SRH less frequently, was mainly performed in our study.

Matsumoto et al. recently reported 2-year outcomes of intravitreous aflibercept therapy for pachychoroid neovasculopathy by treat-and-extend dosing regimen^20^. They showed visual acuity improved rapidly in the first year, and this gain of vision was retained until 2 years. Large scale, long-term, prospective studies are necessary to establish the treatment of pahcychoroid neovasculopathy.

Our findings that eyes with SRH had larger GLD and were more likely to have polypoidal lesions are similar to those found in eyes with neovascular AMD. Hirami et al. reported that a large PCV lesion increased the risk of serious hemorrhagic complications after PDT monotherapy^19^. Tsujikawa et al. reported that PCV with small vascular lesions showed minimal progression and had no vision-threatening complications, and these eyes often maintained good visual acuity for a long period of time^21^. Kitagawa et al. reported that the underlying cause of submacular hemorrhage was exudative AMD in one eye and PCV in 19 eyes^22^. All these results suggest that a larger lesion and the presence of polypoidal lesions are risk factors for SRH, both in neovascular AMD and pachychoroid neovasculopathy. Sakurada et al. showed that *ARMS2* A69S variants were significantly associated with higher incidences of subretinal hemorrhage, serous pigment epithelial detachment (PED), and hemorrhagic PED^23^. However, there were no significant differences in the frequency of these alleles between the SRH group and the non-SRH group in pachychoroid neovasculopathy. There was no significant difference of GLD in Polyp (+) group and in Polyp (−) group. While more SRH (≥ 4 disc areas in size) were detected in Polyp (+) group than in Polyp (−) group, some cases had relatively small lesion size regardless of having polypoidal lesion.

The present study showed that the factors most significantly associated with final BCVA were initial BCVA and the presence of SRH in pachychoroid neovasculopathy. Other research groups have reported that visual acuity in eyes with neovascular AMD at baseline is associated with visual acuity at the final visit^24,25^. In addition, it has been reported that SRH is closely associated with loss of visual function in neovascular AMD^24^. Scupola et al. reported that the visual outcome in eyes with submacular hemorrhages arising from AMD was very poor^25^. SRH is associated with the final BCVA, so prompt treatment, such as gas injection, is necessary when SRH occurs to avoid a poor visual outcome in pachychoroid neovasculopathy as well as in neovascular AMD. Kitagawa et al. recently reported that intravitreal injection of the recombinant tissue plasminogen activator, ranibizumab, along with gas is useful to displace the hemorrhage and improve the lesion in eyes with SRH^22^. Kadonosono et al. reported that submacular injection of air with a microneedle facilitates displacement of clots, leading to earlier visual improvement^26^.

Genetic analysis in our study showed the frequency of the T risk allele of *ARMS2* A69S (46.7%), the A risk allele of *CFH* I62V (25.5%), and the C risk allele of *CFH* Y402H (10.9%) in patients with pachychoroid neovasculopathy. Miyake et al. reported that the frequencies of the T allele in *ARMS2* A69S was 51.3% in patients with pachychoroid neovasculopathy^6^. The prevalence of the T allele in *ARMS2* A69S in the current study is comparable with that reported by Miyake et al. and similar to that in normal Japanese subjects^27^. Dansingani et al. reported on the risk alleles associated with neovascularization in patients with the pachychoroid phenotype and showed that the frequencies of the T allele in *ARMS2* A69S and of the C allele in *CFH* Y402H were 41% and 46%, respectively^28^. The discrepancy in the C allele in *CFH* Y402H may reflect a genetic difference between the Japanese and Caucasian races. It has been reported that the C allele in *CFH* Y402H is rare in Asians^29^. In this study, the frequency of the A allele in *CFH* I62V is 25.5% in pachychoroid neovasculopathy. Hosoda et al. reported major / minor allele (G/A) was 0.464 in eyes with central serous chorioretinopathy, a phenotype of pachychoroid diseases^30^. This discrepancy may be due to the small sample size of pachychoriod neovasculopathy, or the difference of central serous chorioretinopathy and pachychoroid neovasculopathy. In view of all these results, it is possible that an as yet unknown genomic factor may be associated with pachychoroid neovasculopathy. Further research, including genome-wide association studies, is needed to identify disease-associated loci in pachychoroid neovasculopathy.

Recently Spaide reported newly recognized form of drusen, named pachydrusen which show different distribution and thicker choroid compared with soft drusen^31^. Drusen-like deposits, which exhibit similar characteristic to pachydrusen, were also reported. The strict definition of pachydrusen / drusen-like deposits has not been established yet, and it is sometimes difficult to distinguish them from soft drusen. In this current study, to increase the specificity of pachychoroid neovasculopathy, we excluded patients with any kind of drusen. However, we need further investigation for pachydrusen-related pachychoroid neovasculopathy.

This study has several limitations. In addition to its retrospective nature and lack of controls, the criteria for diagnosing pachychoroid neovasculopathy may not be ideal because no standard diagnostic criteria have been established thus far. However, we have revised the definition criteria for pachychoroid neovasculopathy according to the most recent research^15^. Other limitations are variable follow-up period, non-standardized treatment strategy, difficulty of detecting the polyps in the presence of SRH at the initial visit or during the follow-up period. The strength of this study is the larger sample size, relatively long-term follow-up, and availability of genetic information in most cases.

In conclusion, we have investigated the clinical and genetic characteristics of patients with pachychoroid neovasculopathy. There was much variation in lesion size and visual function in patients with this condition. Eyes with SRH had a poor visual prognosis and polypoidal lesions were associated with occurrence of SRH in pachychoroid neovasculopathy.

## Methods

The study was an interventional case-series, and conducted in accordance with the tenets of the Declaration of Helsinki and approved by the Institutional Review Board and Ethics Committee of Kyoto University Graduate School of Medicine. Written informed consent was obtained from each genotyped patient.

### Participants

We retrospectively reviewed the medical records of consecutive patients with treatment-naïve pachychoroid neovasculopathy who initially visited the Macular Service at Kyoto University Hospital between January 2010 and May 2015 and were followed up for more than one year. In this study, the criteria for a diagnosis of pachychoroid neovasculopathy were as follows^15^: Choroidal neovascuralization in either eye; no drusen or only non-extensive hard drusen (≤ 63 μm) in both eyes (Age-Related Eye Disease Study level 1^31^, no AMD); and clinical features of the pachychoroid phenotype, i.e., reduced fundus tessellation on color fundus photographs, dilated outer choroidal vessels on optical coherence tomography (OCT) and indocyanine green angiography (IA) images^8^, and/or regional CVH on IA images. Dilated choroidal vessels were defined as pathologically dilated outer choroidal vessels with attenuation of the choriocapillaris on OCT^8^. On IA, the dilated choroidal vessels were seen to extend from 1 or more vortex vein ampullas^9^. presence of dilated choroidal vessels was judged using the multimodal imaging. A representative eye diagnosed to have pachychoroid neovasculopathy is shown in Figs. 1 and 2. The diagnosis was made by two retinal specialists; if there was discrepancy in diagnosis, a third retinal specialist determined the final diagnosis. If both eyes met the inclusion criteria, only right eye was selected. In this study, eyes with pachydrusen^30^ were not diagnosed as pachychoroid neovasculopathy. Eyes with angioid streaks, uveitis, trauma, other secondary CNV, vitelliform macular dystrophy uveitis, or any history or sign of retinal surgery were excluded, as were eyes with high myopia (axial length ≥ 26.5 mm), diabetic retinopathy, retinal vein occlusion, or other retinal disease. During this target period, 322 eyes had polypoidal lesions at the first visit, and 55 eyes were included as pachychoroid neovasculopathy which satisfied the inclusion criteria and exclusion criteria of this study. History of smoking was asked to each patient and showed in the number of past and current smokers.

### Multimodal imaging

All patient underwent a comprehensive ophthalmologic examination, including BCVA with a Landolt chart, intraocular pressure, indirect ophthalmoscopy, slit-lamp biomicroscopy, automatic objective refraction, axial length measurement (IOL Master 500, Carl Zeiss Meditec, Dublin, CA), color fundus photography, fundus autofluorescence, infrared reflectance, fluorescein angiography (FA), IA, and spectral-domain optical coherence tomography (SD-OCT).

Color fundus photographs (field, 40°) were obtained digitally using a non-mydriatic retinal camera (Topcon, Tokyo, Japan). The fundus autofluorescence, infrared reflectance, FA, and IA images were acquired using a confocal scanning laser ophthalmoscope (Spectralis HRA + OCT; Heidelberg Engineering, Heidelberg, Germany). The field of view was set to 30° × 30° centered on the macula. SD-OCT was performed using Spectralis (Heidelberg Engineering). First, horizontal and vertical line scans through the fovea center were obtained at a 30° angle, followed by serial horizontal scans (30° × 10°). Inverted OCT images were routinely obtained in all patients using an enhanced-depth imaging technique^33^. Fifty SD-OCT images were averaged to reduce speckle noise.

### Image analysis

Drusen were graded based on the fundus photographs according to the grading system for classifying AMD from the Age-Related Eye Disease Study^32^ and confirmed by SD-OCT. In the SD-OCT images, CRT was defined as the mean distance between the vitreoretinal surface and retinal pigment epithelium within a 1-mm circle from the center of the fovea, which was calculated automatically. Subfoveal choroidal thickness, defined as the distance between Bruch’s membrane and the chorioscleral interface at the fovea, was measured manually in enhanced-depth imaging OCT images using the built-in calipers of the software. Both horizontal and vertical scans including the center of the fovea were used, and the average thickness values were calculated. On FA images, we demarcated the outlines of lesions suggestive of CNV manually and measured the GLD and lesion size using the built-in calipers. GLD was defined as the maximum diameter of lesions including the neovascularization and any lesions which might block neovascularization. The IA images were evaluated for the presence of polypoidal lesions. CVH was evaluated as described elsewhere^15,34,35^. Briefly, CVH was determined by detecting multifocal hyperfluorescent areas with blurred margins that expanded during the late phase of IA and were typically accompanied by punctate hyperfluorescent spots in the center.

### Genotyping

Genotyping was performed in 92 patients. Genomic DNA was prepared from leukocytes of peripheral blood using a DNA extraction kit (QuickGene-610L; Fujifilm, Tokyo, Japan). We genotyped the major AMD-associated single nucleotide polymorphisms, i.e., *ARMS2* A69S rs10490924, *CFH* I62V rs800292, and *CFH* Y402H rs1061170, using TaqMan ANP assays with the ABI PRISM 7700 system (Applied Biosystems Inc, Foster City, CA). I

### Treatment

The patients with pachychoroid neovasculopaty were treated with anti-VEGF drugs (e.g.: ranibizumab or aflibercept), or PDT combined with anti-VEGF therapy. Between January 2010 and October 2012, patients were treated with 3 loading intravitreal injections of 0.5 mg ranibizumab at 1-month intervals. After the 3 loading injections, patients were followed up every month, and retreatments were performed as required when VA declined more than 0.2 logMAR along with signs of exudation on OCT or angiography, when retinal thickness increased greater than 100 µm, or if subretinal fluid, subretinal hemorrhage, or active CNV persisted or developed^36^. PDT combined therapy was administered to those resistant to ranibizumab eyes whose retinal edema or subretinal fluid did not decrease after initial ranibizumab treatment. Between November 2012 and May 2015, patients underwent 3 courses of monthly injections and subsequent bi-monthly injections of aflibercept (2.0 mg). The number of injection should be 3 + 4 in the 12-month study period. When there was a contraindication such as cerebral infarction or when patients did not agree to undergo the treatment, injections was skipped. Alternatively, additional injections were administered or PDT combined therapy was performed at the physicians’ discretion^37^.

### Statistical analysis

All data are presented as the mean ± standard deviation. For the statistical analysis, the measured BCVA was converted to logMAR units. The independent samples *t*-test was used to compare variables between the initial condition and the final condition. The paired-samples *t*-test was used to compare mean BCVA and CRT. The unpaired-samples *t*-test was used to compare variables between eyes with and without SRH and polypoidal lesion. The χ^2^ test was used for categorical analysis. The genotype distribution was compared using the χ^2^ test for trends. All statistical evaluations were performed using SPSS version 22 software (IBM Corp., Armonk, NY). A *P*-value less than 0.05 was considered to be statistically significant.
